# The Kinetics of the Humoral and Interferon-Gamma Immune Responses to Experimental *Mycobacterium bovis* Infection in the White Rhinoceros (*Ceratotherium simum*)

**DOI:** 10.3389/fimmu.2017.01831

**Published:** 2017-12-22

**Authors:** Sven D. C. Parsons, Darshana Morar-Leather, Peter Buss, Jennifer Hofmeyr, Ross McFadyen, Victor P. M. G. Rutten, Paul D. van Helden, Michele A. Miller, Anita Luise Michel

**Affiliations:** ^1^DST/NRF Centre of Excellence for Biomedical Tuberculosis Research, Cape Town, South Africa; ^2^SAMRC Centre for TB Research, Cape Town, South Africa; ^3^Division of Molecular Biology and Human Genetics, Faculty of Medicine and Health Sciences, Stellenbosch University, Cape Town, South Africa; ^4^Bovine Tuberculosis and Brucellosis Research Programme, Faculty of Veterinary Science, Department of Veterinary Tropical Diseases, University of Pretoria, Onderstepoort, South Africa; ^5^Veterinary Wildlife Services, South African National Parks, Kruger National Park, Skukuza, South Africa; ^6^Centre for Proteomic and Genomic Research (CPGR), Cape Town, South Africa; ^7^Faculty of Veterinary Medicine, Department of Infectious Diseases and Immunology, Utrecht University, Utrecht, Netherlands; ^8^National Zoological Gardens of South Africa, Pretoria, South Africa

**Keywords:** immune response, interferon-gamma, serology, *Mycobacterium bovis*, tuberculosis, white rhinoceros

## Abstract

*Mycobacterium bovis* is the cause of tuberculosis (TB) in a wide range of species, including white rhinoceroses (*Ceratotherium simum*). Control of the disease relies on the indirect detection of infection by measuring pathogen-specific responses of the host. These are poorly described in the white rhinoceros and this study aimed to characterize the kinetics of immune responses to *M. bovis* infection in this species. Three white rhinoceroses were infected with *M. bovis* and their immune sensitization to this pathogen was measured monthly for 20 months. Cell-mediated immunity was characterized in whole blood samples as the differential release of interferon-gamma in response to bovine purified protein derivative (PPDb) and avian PPD (PPDa) as well as the release of this cytokine in response to the *M. bovis* proteins 6 kDa early secretory antigenic target (ESAT-6)/10 kDa culture filtrate protein (CFP-10). Humoral immunity was quantified as the occurrence or the magnitude of antibody responses to the proteins ESAT-6/CFP-10, MPB83, MPB83/MPB70, and PPDb. The magnitude and duration of immune reactivity varied between individuals; however, peak responses to these antigens were detected in all animals circa 5–9 months postinfection. Hereafter, they gradually declined to low or undetectable levels. This pattern was associated with limited TB-like pathology at postmortem examination and appeared to reflect the control of *M. bovis* infection following the development of the adaptive immune response. Measurement of these markers could prove useful for assessing the disease status or treatment of naturally infected animals. Moreover, immune responses identified in this study might be used to detect infection; however, further studies are required to confirm their diagnostic utility.

## Introduction

White rhinoceroses (*Ceratotherium simum*) are classified as “Near Threatened” by the International Union for Conservation of Nature, with the majority of animals occurring in South Africa (1). Of these, a substantial number occur in the greater Kruger National Park (KNP) and the Hluhluwe-iMfolozi Park (HiP). However, because of their economic value and threatened conservation status, animals from these populations are regularly translocated to other reserves and privately owned collections. Importantly, movement of animals from these areas could present a risk of translocating *Mycobacterium bovis*, a major cause of tuberculosis (TB). This pathogen can infect a wide variety of domestic and wildlife hosts, including rhinoceros species (2) and has become established in both the KNP and HiP (3). The recent detection, in the KNP, of a case of severe pulmonary TB in a black rhinoceros (4) and confirmed *M. bovis* granulomas in lymph nodes of four white rhinoceroses (unpublished data) highlights the potential risk of movement of these species.

Tuberculosis is slowly progressive and the causative organisms may initially be contained within well circumscribed granulomas (5). For this reason, detection of the pathogen can be challenging and, as in other species, TB in rhinoceroses might only be diagnosed postmortem or once animals have developed advanced disease (2). Infection is therefore often diagnosed indirectly by measuring the host’s adaptive immune response toward *M. bovis* antigens. This is commonly done by quantifying either the *in vivo* or the *in vitro* immune response to purified protein derivative (PPD), a preparation containing a broad range of *M. bovis* antigens (6, 7). Alternatively, recombinant proteins that are more specific to *M. bovis* can be utilized as test antigens and these include 6 kDa early secretory antigenic target (ESAT-6), 10 kDa culture filtrate protein (CFP-10), MPB70, and MPB83 (8). Assessment of immune responses to these and other antigens might also be used to distinguish between latent infection and progressive disease or to monitor treatment in both humans and animals (8, 9).

The present study forms part of a broader project that characterized the clinical features and associated gross and histopathology of the experimental infection of three white rhinoceroses with *M. bovis* (10). In all cases, 20 months after infection, animals had shown no clinical signs of TB disease and had limited TB-like pathology (10). We hypothesized that quantifying the adaptive immune responses to *M. bovis* in these animals would provide an indirect measure of their infection and disease status. As such, we aimed to characterize (i) their immune sensitization to selected antigens following infection and (ii) the kinetics of their humoral and cell-mediated immune responses.

## Materials and Methods

### Animals

The capture, maintenance, chemical immobilization, infection, and sampling of animals in the KNP, as well as biohazard containment, have previously been described in detail (10). Briefly, three subadult male white rhinoceroses, identified as PB1, PB2, and PB4, were infected by endoscopic endobronchial instillation of the *M. bovis* strain SB0121, a genotype commonly isolated from wildlife in the KNP. The inocula for the three animals contained approximately 2.1 × 10^3^ colony forming units (cfu), 1.8 × 10^2^ and 1.4 × 10^3^ cfu, respectively. Each month, from 3 months prior to infection until 20 months postinfection (PI), animals were chemically immobilized and blood was collected from the radial vein into lithium heparin and serum vacutainer tubes (Fisher Scientific, Suwanee, GA, USA) and used in the immunological assays described below (Table 1). On these occasions, endoscopic bronchoalveolar lavages were performed for mycobacterial culture. Twenty months after infection, animals were euthanized, postmortem examinations performed, and systematic tissue sampling conducted to determine the presence or absence of *M. bovis* by histopathology, mycobacterial culture, and polymerase chain reaction (PCR) and findings have been previously reported in detail (10). Approval for the study was obtained from the Animal Ethics Committees of the South African National Parks and Stellenbosch University (proposal SU-ACUM12-00012) as well the South African National Department of Agriculture, Forestry and Fisheries in terms of Section 20 of the Animal Diseases Act (Permit 12/11/1/1/6/1).

**Table 1:** Immunoassays utilized to measure immune sensitization to selected antigens in *Mycobacterium bovis*-infected white rhinoceroses.

Assay	Supplier	Test antigens	Assay format	Reference
Rhinoceros-specific assay	In-house assay	*M. bovis* PPD; *M. avium* PPD	IGRA	(11)
Modified QFT assay	Components supplied by Qiagen and Mabtech	ESAT-6, CFP-10, TB 7.7	IGRA	N/A
PPD ELISA	In-house assay	*M. bovis* PPD	ELISA	N/A
ElephantTB STAT-PAK^®^ assay	Chembio Diagnostic Systems	ESAT-6/CFP-10/MPB83	LFD	(8, 12)
Dual Path Platform (DPP)^®^ VetTB assay	Chembio Diagnostic Systems	MPB83; ESAT-6/CFP-10	LFD	(12)
Bovid DPP assay	Chembio Diagnostic Systems	MPB83/MPB70	LFD	N/A

*PPD, purified protein derivative; IGRA, interferon-gamma release assay; QFT, QuantiFERON TB Gold (In Tube); N/A, not applicable; ELISA, enzyme-linked immunosorbent assay; LFD, lateral flow device*.

### Rhinoceros-Specific Interferon-Gamma (IFN-γ) Release Assay

Blood collected in heparin tubes was processed within 12 h after collection as previously described (11). Briefly, whole blood samples were incubated at 37°C in 5% CO_2_ for 24 h with *M. bovis* PPD (PPDb, 20 µg/ml) and *M. avium* PPD (PPDa, 20 µg/ml), PMA/CaI (0.1/2 μg/ml—positive control), culture medium (Nil—negative control), respectively. Plasma was harvested following centrifugation at 1,088 × *g* for 5 min and stored at −80°C until tested, in duplicate, in the rhinoceros-specific IFN-γ capture enzyme-linked immunoassay (ELISA) (11). The results of the ELISA were determined at 490 nm using an ELISA plate reader (BioTek, Powerwave XS2, Gen5 software). An initial reference/blank reading was performed on the plate prior to the blocking step, at the same wavelength, and final optical density (OD) values were determined by subtracting the mean reference value from the mean test value for each sample well. Antigen- and mitogen-specific release of IFN-γ was calculated as the OD value derived from the PPD- and PMA/CaI-stimulated samples minus that derived from the Nil sample. *M. bovis*-specific release of IFN-γ was calculated as the PPDb value minus the PPDa value (ΔPPDb-a).

### Modified QuantiFERON TB Gold (In-Tube) (QFT) Assay

One milliliter of heparinized whole blood was transferred to a “Nil” tube (containing saline) and a “TB Antigen” tube (coated with peptides simulating ESAT-6, CFP-10, and TB 7.7) of the QFT system (Qiagen, Hilden, Germany). In addition, as a measure of cell viability, 1 ml blood was incubated with phytohemagglutinin (PHA) (Sigma-Aldrich, St. Louis, MO, USA) in phosphate-buffered saline (PBS) at a final concentration of 10 µg/ml. The tubes were shaken according to the manufacturer’s instructions and incubated for 20–24 h at 37°C. Hereafter, the tubes were centrifuged at 1,600 × *g* for 10 min and plasma was harvested and stored at −80°C. Plasma samples were assayed in duplicate using a commercial bovine IFN-γ ELISA cross-reactive with IFN-γ of sheep and horses (Kit 3115-1H-20; Mabtech AB, Nacka Strand, Sweden) that has previously been shown to detect recombinant rhinoceros IFN-γ (data not shown). Reactions were visualized using 3,3′,5,5′-tetramethylbenzidine (TMB) (BD Biosciences, NJ, USA) as a color substrate. The IFN-γ concentration in each sample was measured as the OD of each well, at a wavelength of 450 nm, using a Labtech LT-4000 microplate reader (Lasec, Cape Town, South Africa). The *M. bovis*-specific release of IFN-γ was calculated as the mean OD derived for plasma harvested from the TB Ag tube minus the mean OD derived for plasma harvested from the Nil tube.

### PPD ELISA

Flat-bottomed 96-well microtiter plates (Nunc, New York, NY, USA) were coated with 100 µl of a 10 µg/ml PPDb solution (Prionics, Schlieren-Zurich, Switzerland) in 0.05 M carbonate-bicarbonate buffer (pH 9.6) and incubated overnight at 4°C. For each well coated with antigen, a corresponding control well was coated with 100 µl blocking buffer (BB) consisting of 5% milk powder (Clover, Roodepoort, South Africa) in PBS + 0.05% Tween-20 (Sigma-Aldrich, St. Louis, MO, USA). After incubation, plates were decanted and washed five times with PBS containing 0.05% Tween-20, then blocked with 200 μl/well of BB for 1 h at room temperature (RT). The plates were washed as above and serum samples diluted 1:200 in BB were added to duplicate wells (100 μl/well). Plates were incubated at RT for 1 h and then washed five times as above. Plates were then incubated with 100 μl/well of peroxidase-conjugated recombinant protein A/G (Thermo Scientific, MA, USA) diluted 1:100,000 in PBS, for 1 h at RT. Plates were washed as above before addition of 100 μl/well of TMB and subsequently incubated in the dark for 15 min. The reaction was stopped using 50 μl/well of 2 M H_2_SO_4_ and the OD of each well was measured at 450 nm using a LT-4000 Microplate Reader (Lasec). For each animal, an assay result was calculated as the mean OD value derived from duplicate PPD-coated wells minus that of duplicate BB-coated wells.

### ElephantTB STAT-PAK^®^ Assay

Rhinoceros sera were tested using the ElephantTB STAT-PAK^®^ assay (Chembio Diagnostic Systems, Inc., Medford, NY, USA). The assay has been optimized for the detection of *M. tuberculosis* infection in elephants but is not specific for a particular host species and has previously been found useful in measuring antibody responses in a black rhinoceros infected with *M. tuberculosis* (12). Briefly, 30 µl of serum was added to the sample well followed by three drops of diluent/antibody detection conjugate. If the sample contained antibodies to *M. bovis* antigens (ESAT-6/CFP-10/MPB83), a positive line appeared as a blue band. Any visible band observed in the test line area by two independent observers and read at 20 min was considered as an antibody positive result.

### Dual Path Platform (DPP)^®^ VetTB and Bovid DPP Assays

The DPP^®^ VetTB and Bovid DPP assays (Chembio Diagnostic Systems, Inc.) were performed according to the manufacturer’s instructions as previously described (12). After 15 min, the test results were read. A DPP optical reader device (Chembio Diagnostic Systems, Inc.) was used to measure the reflectance of test strips and a result was quantified as a numerical score, represented as reflectance units (RU). The DPP^®^ VetTB assay includes two test lines containing the antigens MPB83 and ESAT-6/CFP-10, along with a positive control. In the Bovid DPP assay, a single test line contained MPB83/MPB70, along with a positive control.

## Results

### Rhinoceros-Specific IFN-γ Assay

**Figure 1:**
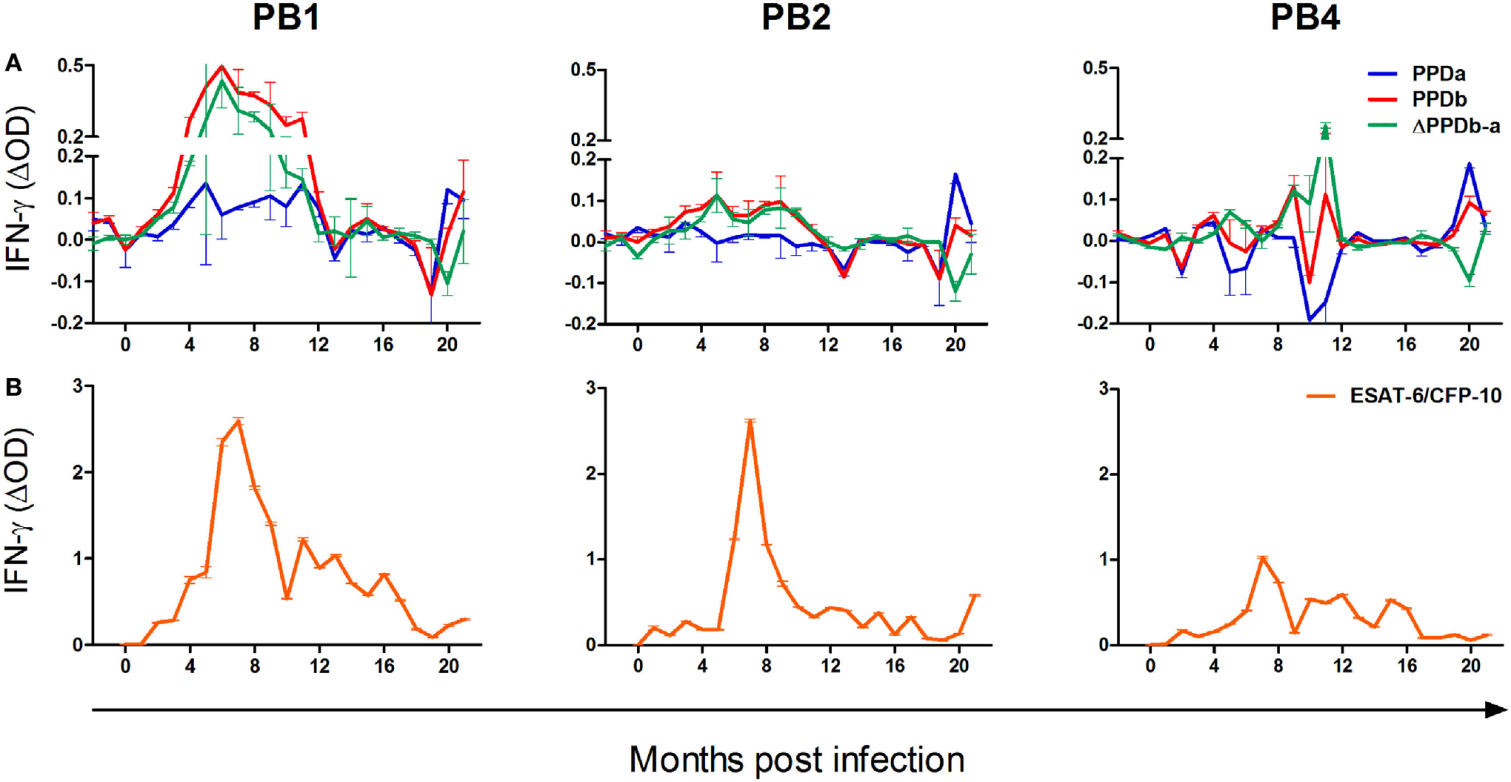
The kinetics of the cell-mediated immune response to *Mycobacterium bovis* infection in the white rhinoceros. Following *M. bovis* infection, whole blood from three animals (PB1, PB2, and PB4) was incubated overnight at 37°C without stimulation, or stimulation with bovine PPD purified protein derivative (PPDb), avian PPD (PPDa), and 6 kDa early secretory antigenic target (ESAT-6)/10 kDa culture filtrate protein (CFP-10)/TB7.7 peptides, respectively. Plasma interferon-gamma (IFN-γ) was measured by enzyme-linked immunoassay, in duplicate, as the optical density (OD), and antigen-specific release calculated as the OD derived for the unstimulated sample subtracted from that derived for each antigen (mean ΔOD ± SD). **(A)** Antigen-specific IFN-γ release in response to PPDb and PPDa, and the differential response to these antigens (ΔPPDb-a); **(B)** Antigen-specific IFN-γ release in response to ESAT-6/CFP-10/TB7.7 peptides.

The release of IFN-γ in unstimulated blood was negligible, resulting in median ELISA OD values of 0.08, 0.05, and 0.07 for PB1, PB2, and PB4, respectively. All animals displayed strong IFN-γ responses to PMA/CaI stimulation, with mean mitogen-specific OD values of 0.86, 0.83, and 0.86, respectively. The kinetics of PPDa and PPDb-specific IFN-γ release in whole blood from these animals are illustrated in Figure 1A. Immune sensitization to these antigens was negligible prior to infection and first observed at 2–3 months PI. The release of IFN-γ in response to both antigens was greatest between 4 and 10 months PI and the *M. bovis*-specific PPD responses (ΔPPDb-a) peaked at circa 5–6 months PI in PB1 and PB2 and at 11 months PI in PB4. Subsequently, IFN-γ release in responses to both PPDa and PPDb were low until 20 months PI when all animals again showed substantial responses to these antigens. At this time, IFN-γ release was notably greater in response to PPDa than PPDb in PB2 and PB4, while in PB1, the PPDb response was higher than that to PPDa.

### Modified QFT Assay

The release of IFN-γ in unstimulated blood was negligible, resulting in median ELISA OD values of 0.09, 0.08, and 0.09 for PB1, PB2, and PB3, respectively. As a result of experimental error, measurements of PHA-induced IFN-γ responses were available for only 14/21 sampling occasions for each animal. On these occasions, median mitogen-specific OD values were 0.81, 0.35, and 0.89, respectively. The kinetics of ESAT-6/CFP-10/TB7.7-specific IFN-γ release in whole blood from PB1, PB2, and PB4 are illustrated in Figure 1B. These antigen-specific responses were first observed at 1 month PI in PB2 and at 2 months PI in PB1 and PB4. Following infection, peak responses occurred at circa 6–9 months PI, after which IFN-γ release decreased gradually over time. At 20 months PI, PB2 displayed a moderate but distinct increase in IFN-γ release in response to ESAT-6/CFP-10/TB7.7.

### PPD ELISA, ElephantTB STAT-PAK^®^, and DPP Assays

**Figure 2:**
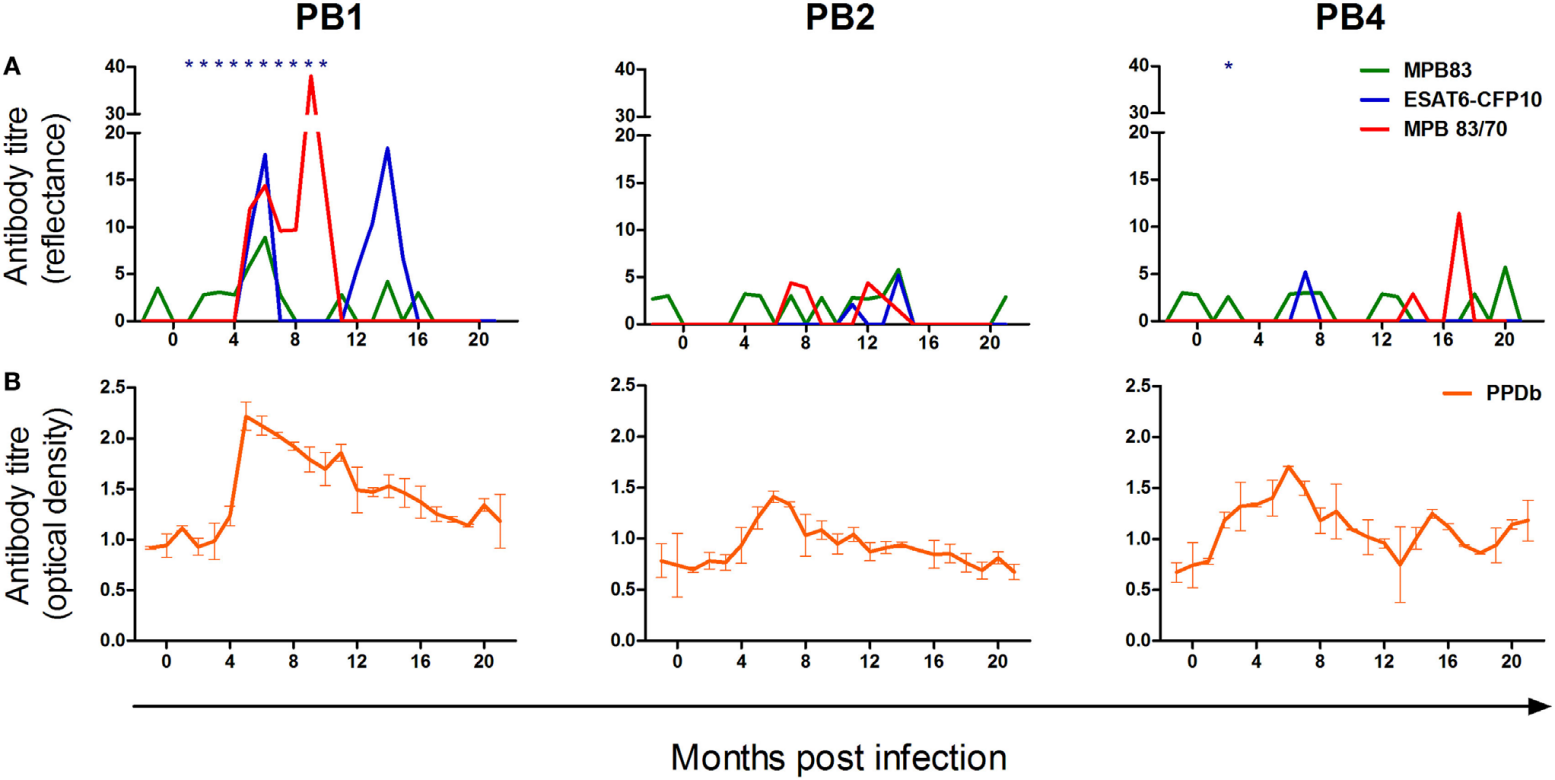
The kinetics of the humoral response to *Mycobacterium bovis* infection in the white rhinoceros. Following *M. bovis* infection, serum antibodies of three animals (PB1, PB2, and PB4) were measured using selected commercial assays and an in-house bovine purified protein derivative (PPDb) enzyme-linked immunoassay (ELISA). **(A)** Serological responses to MPB83 and 6 kDa early secretory antigenic target (ESAT-6)/10 kDa culture filtrate protein (CFP-10) (Dual Path Platform (DPP)^®^ VetTB assay) and MPB83/70 (Bovid DPP assay); seroreactivity to ESAT-6/CFP-10/MPB83 (ElephantTB STAT-PAK^®^ assay) is indicated (*). **(B)** Serological responses to PPDb, measured in duplicate by indirect ELISA, and calculated as the difference in optical density (OD) between control wells and PPDb-coated wells (mean ΔOD ± SD).

The kinetics of the humoral responses to *M. bovis* antigens in PB1, PB2, and PB4 are illustrated in Figure 2. Using the ElephantTB STAT-PAK^®^ assay, PB1 displayed humoral sensitization to the combined antigens ESAT-6/CFP-10/MPB83 at all time points from 1 to 10 months PI (Figure 2A). In contrast, antibodies to this antigen pool were not detected in PB2 for the duration of the study and only on a single occasion in PB4, i.e., 2 months PI (Figure 2A). Using the DPP assays, only PB1 displayed a strong and sustained humoral response to the *M. bovis* antigens ESAT-6/CFP-10, MPB83, and MPB83/MPB70, with sensitization to all three antigens detected at 5 and 6 months PI. Hereafter, the humoral response to MBP83 alone was not again detected in this animal; however, responses to MBP83/MPB70 were sustained until 10 months PI and responses to ESAT-6/CFP-10 peaked again at 14 months PI. Both PB2 and PB4 displayed very low antibody quantities specific for these antigens, with PB4 showing only a moderate response to MPB83/MPB70 at 17 months PI.

Prior to infection, levels of circulating antibodies to PPDb (Figure 2B) were similar in all animals, with levels rising from 2 to 4 months PI. A peak in the PPDb-specific humoral response occurred circa 6 months PI, after which this gradually decreased in all animals.

## Discussion

Following experimental endobronchial instillation of *M. bovis* in 3 white rhinoceroses, these developed time-dependent humoral and cell-mediated immune responses toward the antigens PPDa, PPDb, ESAT-6/CFP-10, MPB83, MPB83/MPB70, and ESAT-6/CFP-10/MPB83. Notably, cell-mediated responses toward ESAT-6/CFP-10 and PPD showed greater consistency between individuals than did humoral responses toward MPB83, MPB83/MPB70, and ESAT-6/CFP-10/MPB83. While the specific patterns of immune reactivity varied between individuals, peak responses were detected in all three animals circa 5–9 months PI, after which they gradually declined to low or undetectable levels.

In mice, the onset of adaptive immunity toward *M. tuberculosis* requires the active replication of the pathogen in lymph nodes draining the site of infection (13). Moreover, the progression and magnitude of this response is associated with the amount of antigen produced in these lymph nodes (13, 14). Similarly, *M. bovis* infection in cattle and deer invariably results in the development of granulomatous disease in lymph nodes draining the site of infection, suggesting that these tissues are the primary site of antigen presentation and immune activation (15). In the present study, during the first 5–9 months PI, the rhinoceroses displayed a consistent rise in immune reactivity toward *M. bovis* antigens, strongly suggesting the establishment of this infection in all three animals.

The magnitude and duration of *M. bovis*-specific immune reactivity appeared to reflect the disease status of each animal. PB1, which displayed the most robust and sustained responses to all test antigens was the only animal from which viable *M. bovis* was isolated, i.e., from a bronchoalveolar lavage sample collected 5 months following infection (10). Moreover, at postmortem examination of PB1, acid fast bacterial rods and *M. bovis* DNA were detected in tracheobronchial and lung lesions, respectively (10). In contrast, PB4 displayed substantially lower responses of both humoral and cell-mediated immunity (CMI) and no definitive confirmation of *M. bovis* infection was made in this animal by culture, histopathology, or PCR (10).

Following an initial peak circa 5–9 months PI, all rhinoceroses displayed a gradual decline in the magnitude of their immune responses, and antigen-induced IFN-γ release reached low levels within 12–16 months after infection. In cattle, the severity of TB disease correlates with both the magnitude of ESAT-6-specific CMI (5) and the humoral response to *M. bovis* (6). Similarly, in rabbits experimentally infected with *M. tuberculosis*, progressive disease is associated with strongly rising titers of anti-PPD antibodies (16), while control of the infection is characterized by low seroreactivity to PPD that declines over time (17). A similar pattern of waning sensitization to ESAT-6/CFP-10 has been documented in naturally infected black rhinoceroses during treatment for *M. tuberculosis* infection (8). Such patterns are associated with a decrease in pathogen burden and antigenic load (17). Notably, for PB1, the peak in CMI responses at 5 months PI occurred at the only time point that viable *M. bovis* was isolated from the respiratory tract. Hereafter, the gradual decline in *M. bovis*-specific immune responses, as for the other two experimentally infected animals, was associated with limited TB-like pathology at postmortem examination and a failure to isolate viable organisms from multiple tissues. Together, these immune response patterns appear to reflect the control of *M. bovis* infection in these animals following the development of the adaptive immune response.

Humoral responses broadly mirrored those of CMI, with PB1 showing strong seroreactivity to all test antigens. However, PB2 and PB4 showed limited detectable sensitization to the *M. bovis*-specific antigens MPB83, MPB83/MPB70, and ESAT-6/CFP-10. Similar differences in seroreactivity of individual animals are seen following experimental infection in other species, including cattle and badgers (18, 19). As for these hosts, this phenomenon in rhinoceroses could be related to the infection dose administered to each animal or reflect a natural animal-to-animal diversity in antigen recognition (18, 19). Nonetheless, following infection, PB2 and PB4 did display a transient elevation in seroreactivity to PPDb. In part, this may reflect the far greater range of antigenic epitopes present in PPDb compared to the more specific antigens such as MPB83 and ESAT-6. Alternatively, because PPD includes antigens that are shared between various mycobacterial species (20), the detection of immunological sensitization to PPDb might in part reflect the boosting of immune responses to cross-reactive antigens. Evidence of such cross-reactivity is indicated by the distinct CMI response of PB1 to PPDa following *M. bovis* infection.

At 20 months PI, all rhinoceroses displayed a rapid increase in IFN-γ release in response to the antigens PPDb, PPDa, and ESAT-6/CFP-10/TB7.7. This boosting of the immune response might have reflected acute progression of TB disease in these animals (5); however, this is unlikely given the paucity of *M. bovis*-associated pathology at postmortem examination shortly after this sampling time point (10). Alternatively, this observation may be related to viral pneumonia observed in PB2 and PB4 (10). The immune response associated with acute viral infections can induce non-specific “bystander” activation and proliferation of lymphocytes (21), giving rise to IFN-γ production (22). Notably, at this time, PB2 and PB4 displayed a greater response to PPDa than to PPDb. This may be further evidence that this phenomenon resulted from a non-specific mycobacterial or viral infection rather than an *M. bovis*-specific event.

Results from the present study provide insight into the temporal patterns of immunological responses to *M. bovis* infection in white rhinoceroses. However, limitations in the study design must be considered when extrapolating findings to animals under natural conditions. First, the nature of the study precluded statistical analysis of data, partly because of the limited sample size and partly because of the substantial variation in immune responses between individuals. Unlike studies using inbred species such as experimental mice (13), this limitation is commonly experienced in studies investigating outbred animals, especially wildlife (19). Second, differences in the infective doses administered to each animal may have accounted for the differences in pathological outcomes and immune response profiles and these may not reflect natural infection. Nonetheless, it is notable that while the magnitude of these responses differed between individuals, their kinetics were comparable. Lastly, the three experimentally infected individuals were all relatively young, had been treated for ectoparasites, were generally free from severe stressors and were well fed (10). As such, the immunological profiles described in this study may reflect an optimal outcome of infection and not necessarily a scenario in which these and other factors might affect either disease progression or resolution.

In summary, this study has characterized antigen-specific immune response patterns of white rhinoceroses that appear to reflect the effective control of *M. bovis* infection by this host. Importantly, the measurement of these responses might prove useful for assessing the disease status or response to treatment of naturally infected animals. Moreover, the immunological markers identified in this study might be used for the detection of infection; however, further studies investigating these markers in *M. bovis*-uninfected animals are required to confirm their diagnostic utility.

## Ethics Statement

This study was carried out with the approval of the Animal Ethics Committees of the South African National Parks (SANParks) and Stellenbosch University. Animal care was in accordance with the SANParks Standard Operating Procedures for the Capture, Transportation and Maintenance in Holding Facilities of Wildlife.

## Author Contributions

AM, MM, VR, and PH designed the study; MM, PB, and JH provided animal care and performed chemical immobilization and blood collection; SP, DM-L, JH, RM, and MM performed laboratory assays; data analysis was done by SP, DM-L, VR, PH, MM, and AM; the first draft of the manuscript was written by SP and all authors contributed to editorial revision.

## Conflict of Interest Statement

The research was conducted in the absence of any commercial or financial relationships that could be construed as a potential conflict of interest.

## Abbreviations

CFP-10, 10 kDa culture filtrate protein; ELISA, enzyme-linked immunoassay; ESAT-6, 6 kDa early secretory antigenic target; HiP, Hluhluwe-iMfolozi Park; IFN-γ, interferon-gamma; KNP, Kruger National Park; OD, optical density; PBS, phosphate-buffered saline; PI, postinfection; PPDa, avian purified protein derivative; PPDb, bovine purified protein derivative; QFT, modified QuantiFERON TB Gold (In-Tube) assay; TMB, 3, 3′, 5, 5′- tetramethylbenzidine.

## References

[1] Emslie R Ceratotherium simum. The IUCN Red List of Threatened Species 2012: e.T4185A16980466. (2012). Available from: http://dx.doi.org/10.2305/IUCN.UK.2012.RLTS.T4185A16980466.en

[2] Miller M Michel A van Helden P Buss P. Tuberculosis in rhinoceros: an underrecognized threat? Transbound Emerg Dis (2017) 64(4):1071–8. 10.1111/tbed.12489 26996520

[3] Michel AL Bengis RG Keet DF Hofmeyr M de Klerk LM Cross PC Wildlife tuberculosis in South African conservation areas: implications and challenges. Vet Microbiol (2006) 112:91–100. 10.1016/j.vetmic.2005.11.035 16343819

[4] Miller MA Buss P Van Helden P Parsons SDC. *Mycobacterium bovis* in a free-ranging black rhinoceros, Kruger National Park, South Africa, 2016. Emerg Infect Dis (2017) 23:557–8. 10.3201/eid2303.161622 28221132 PMC5382732

[5] Vordermeier HM Chambers MA Cockle PJ Whelan AO Simmons J Hewinson RG. Correlation of ESAT-6-specific gamma interferon production with pathology in cattle following *Mycobacterium bovis* BCG vaccination against experimental bovine tuberculosis. Infect Immun (2002) 70:3026–32. 10.1128/IAI.70.6.3026-3032.2002 12010994 PMC128013

[6] Welsh MD Cunningham RT Corbett DM Girvin RM McNair J Skuce RA Influence of pathological progression on the balance between cellular and humoral immune responses in bovine tuberculosis. Immunology (2005) 114:101–11. 10.1111/j.1365-2567.2004.02003.x 15606800 PMC1782060

[7] Griffin JFT Nagai S Buchan GS. Tuberculosis in domesticated red deer: comparison of purified protein derivative and the specific protein MPB70 for in vitro diagnosis. Res Vet Sci (1991) 50:279–85. 10.1016/0034-5288(91)90124-7 1882133

[8] Duncan AE Lyashchenko K Greenwald R Miller M Ball R. Application of elephant TB Stat-Pak assay and Mapia (multi-antigen print immunoassay) for detection of tuberculosis and monitoring of treatment in black rhinoceros (*Diceros bicornis*). J Zoo Wildl Med (2009) 40:781–5. 10.1638/2009-0044.1 20063826

[9] Chegou NN Black GF Kidd M van Helden PD Walzl G. Host markers in Quantiferon supernatants differentiate active TB from latent TB infection: preliminary report. BMC Pulm Med (2009) 9:21. 10.1186/1471-2466-9-21 19445695 PMC2696407

[10] Michel AL Lane EP De Klerk-Lorist L-M Hofmeyr M Van der Heijden EMDL Botha L Experimental *Mycobacterium bovis* infection in three white rhinoceroses (*Ceratotherium simum*): susceptibility, clinical and anatomical pathology. PLoS One (2017) 12:e0179943. 10.1371/journal.pone.0179943 28686714 PMC5501512

[11] Morar D Schreuder J Mény M van Kooten PJS Tijhaar E Michel AL Towards establishing a rhinoceros-specific interferon-gamma (IFN-γ) assay for diagnosis of tuberculosis. Transbound Emerg Dis (2013) 60:60–6. 10.1111/tbed.12132 24171850

[12] Miller MA Greenwald R Lyashchenko KP. Potential for serodiagnosis of tuberculosis in black rhinoceros (*diceros bicornis*). J Zoo Wildl Med (2015) 46:100–4. 10.1638/2014-0172R1.1 25831581

[13] Wolf AJ Desvignes L Linas B Banaiee N Tamura T Takatsu K Initiation of the adaptive immune response to *Mycobacterium tuberculosis* depends on antigen production in the local lymph node, not the lungs. J Exp Med (2008) 205:105–15. 10.1084/jem.20071367 18158321 PMC2234384

[14] Reiley WW Calayag MD Wittmer ST Huntington JL Pearl JE Fountain JJ ESAT-6-specific CD4 T cell responses to aerosol *Mycobacterium tuberculosis* infection are initiated in the mediastinal lymph nodes. Proc Natl Acad Sci U S A (2008) 105:10961–6. 10.1073/pnas.0801496105 18667699 PMC2504808

[15] Behr MA Waters WR. Is tuberculosis a lymphatic disease with a pulmonary portal? Lancet Infect Dis (2014) 14:250–5. 10.1016/S1473-3099(13)70253-6 24268591

[16] Subbian S Tsenova L Yang G O’Brien P Parsons S Peixoto B Chronic pulmonary cavitary tuberculosis in rabbits: a failed host immune response. Open Biol (2011) 1:110016. 10.1098/rsob.110016 22645653 PMC3352086

[17] Subbian S Tsenova L O’Brien P Yang G Kushner NL Parsons S Spontaneous latency in a rabbit model of pulmonary tuberculosis. Am J Pathol (2012) 181:1711–24. 10.1016/j.ajpath.2012.07.019 22960076 PMC3483799

[18] Lyashchenko KP Pollock JM Colangeli R Gennaro ML. Diversity of antigen recognition by serum antibodies in experimental bovine tuberculosis. Infect Immun (1998) 66:5344–9. 9784542 10.1128/iai.66.11.5344-5349.1998 PMC108668

[19] Lesellier S Corner L Costello E Sleeman P Lyashchenko KP Greenwald R Immunological responses following experimental endobronchial infection of badgers (*Meles meles*) with different doses of *Mycobacterium bovis*. Vet Immunol Immunopathol (2009) 127:174–80. 10.1016/j.vetimm.2008.09.012 18986710

[20] Michel AL *Mycobacterium fortuitum* infection interference with *Mycobacterium bovis* diagnostics: natural infection cases and a pilot experimental infection. J Vet Diagn Invest (2008) 20:501–3. 10.1177/104063870802000415 18599858

[21] Murali-Krishna K Altman JD Suresh M Sourdive DJD Zajac AJ Miller JD Counting Antigen-Specific CD8 T Cells: a re-evaluation of bystander activation during viral infection. Immunity (1998) 8:177–87. 10.1016/S1074-7613(00)80470-7 9491999

[22] Gilbertson B Germano S Steele P Turner S Fazekas de St Groth B Cheers C. Bystander activation of CD8+ T Lymphocytes during experimental mycobacterial infection. Infect Immun (2004) 72:6884–91. 10.1128/IAI.72.12.6884-6891.2004 15557609 PMC529149

